# Predicting amyloid accumulation in subjects with cognitive disorders by using machine learning with ^18^F‐FDG‐PET images

**DOI:** 10.1002/alz70856_102438

**Published:** 2025-12-25

**Authors:** Kazunari Ishii, Takahiro Yamada, Kohei Hanaoka, Hayato Kaida, Yuichi Kimura

**Affiliations:** ^1^ Kindai University Faculty of Medicine, Osakasayama, Osaka, Japan; ^2^ Kindai University Hospital, Osakasayama, Osaka, Japan; ^3^ Kindai University Graduate School of Science and Engineering, Higashiosaka, Osaka, Japan

## Abstract

**Background:**

Since the approval of disease‐modifying drugs for Alzheimer's disease, the demand for amyloid positron emission tomography (PET) scans, which are crucial for determining treatment eligibility, is expected to increase significantly. We thus investigated the ability of an algorithm to predict amyloid accumulation from ^18^F‐fluorodeoxyglucose (FDG)‐PET images for use in amyloid PET screening.

**Method:**

We analyzed the images of 194 subjects with cognitive disorders who had undergone brain FDG‐PET, amyloid PET using Pittsburgh compound‐B (^11^C‐PiB), and MRI scans at Kindai University Hospital between 2011 and 2018. Among them, 108 subjects showed positive amyloid accumulation; the other 86 did not. For the 108 positive cases, the input values were the region of interest‐based calculated from the automatic anatomical labeling template, which divides the brain into 120 regions, applied to the anatomically standardized FDG‐PET images of each subject. We then used a support‐vector machine (SVM) machine learning algorithm and conducted a 10‐fold cross‐validation to assess the algorithm's accuracy for predicting amyloid accumulation from FDG‐PET images.

**Result:**

We observed 81.5% accuracy, 78.5% sensitivity, 84.6% specificity, and an area under the curve (AUC) of 0.846 during training. The validation results for the trained model revealed 85.9% accuracy, 88.4% sensitivity, 81.0% specificity, and an AUC of 0.918.

**Conclusion:**

The performance of our algorithm to predict amyloid accumulation in subjects with cognitive disorders from ^18^FDG‐PET images is adequate for use in amyloid PET scan screenings.

